# Quality Assessment and Morphological Analysis of Photoplethysmography in Daily Life

**DOI:** 10.3389/fdgth.2022.912353

**Published:** 2022-07-07

**Authors:** Serena Moscato, Luca Palmerini, Pierpaolo Palumbo, Lorenzo Chiari

**Affiliations:** ^1^Department of Electrical, Electronic, and Information Engineering “Guglielmo Marconi” – DEI, University of Bologna, Bologna, Italy; ^2^Health Sciences and Technologies-Interdepartmental Center for Industrial Research (CIRI-SDV), University of Bologna, Bologna, Italy

**Keywords:** photoplethysmography, quality assessment, wearable device, morphological analysis, pervasive monitoring

## Abstract

The photoplethysmographic (PPG) signal has been applied in various research fields, with promising results for its future clinical application. However, there are several sources of variability that, if not adequately controlled, can hamper its application in pervasive monitoring contexts. This study assessed and characterized the impact of several sources of variability, such as physical activity, age, sex, and health state on PPG signal quality and PPG waveform parameters (Rise Time, Pulse Amplitude, Pulse Time, Reflection Index, Delta T, and DiastolicAmplitude). We analyzed 31 24 h recordings by as many participants (19 healthy subjects and 12 oncological patients) with a wristband wearable device, selecting a set of PPG pulses labeled with three different quality levels. We implemented a Multinomial Logistic Regression (MLR) model to evaluate the impact of the aforementioned factors on PPG signal quality. We then extracted six parameters only on higher-quality PPG pulses and evaluated the influence of physical activity, age, sex, and health state on these parameters with Generalized Linear Mixed Effects Models (GLMM). We found that physical activity has a detrimental effect on PPG signal quality quality (94% of pulses with good quality when the subject is at rest vs. 9% during intense activity), and that health state affects the percentage of available PPG pulses of the best quality (at rest, 44% for healthy subjects vs. 13% for oncological patients). Most of the extracted parameters are influenced by physical activity and health state, while age significantly impacts two parameters related to arterial stiffness. These results can help expand the awareness that accurate, reliable information extracted from PPG signals can be reached by tackling and modeling different sources of inaccuracy.

## Introduction

The digital healthcare revolution promises to switch from a hospital-centered model to a personal-centered model (1), offering the possibility to remotely and continuously monitor patients' health state, thus reducing the use of bulky instruments and complicated procedures (2). One of the key elements of this revolution is represented by wearable devices, which are small electronic systems that can be worn during daily life (3). However, such devices are not used as diagnostic tools yet for several reasons, including ethical aspects, limitations in the infrastructure, and concerns related to data protection (4). Nonetheless, wearable sensors have been used in several applications for research purposes, ranging from rehabilitation (4) and sport (5) to cardiovascular monitoring (6) and emotion recognition (7).

**Figure 1:**
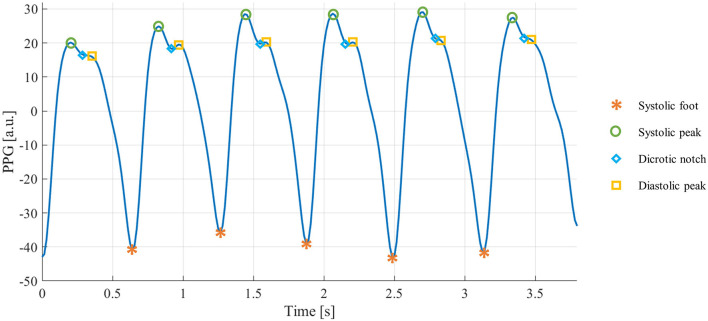
PPG signal and highlighted fiducial points.

Photoplethysmographic (PPG) sensors are one of the most widespread technologies within wearable devices (8). They are based on an optical technique: a light source illuminates a portion of the body surface, penetrating the skin and reaching the blood vessels, and a matched photodetector detects the changes modulated by the pulsatile component of the blood flow (9). The resulting signal is quasi-periodic, consisting of a stereotyped waveform called “PPG pulse,” which occurs with each heartbeat (10). Each PPG pulse contains several fiducial points, each of them corresponding to a cardiac event (11), as shown in Figure 1:

- Systolic foot: represents the minimum of the pulse and corresponds to the beginning of the systolic phase of the heart;
- Systolic peak: represents the maximum of the pulse and corresponds to the maximum blood volume during the systolic phase of the heart;
- Dicrotic notch: a local minimum corresponding to the aortic valve closure;
- Diastolic peak: represents the second maximum of the pulse and corresponds both to the diastolic phase of the heart and wave reflection in the periphery.

For various reasons, the fiducial points are not always traceable in the PPG pulses. Based on the fiducial points that can be detected, the quality of each PPG pulse can be expressed as (12):

- Basic quality: systolic peaks are identifiable, so reliable heart rate, heart rate variability parameters, and some basic morphological features can be derived;
- Diagnostic quality: systolic and diastolic peaks are visible, so a more in-depth morphological analysis can be conducted.

Currently, PPG sensors are mainly used for heart rate estimation in a real-world context: the heart rate can be estimated by simply calculating the time distance between two consecutive systolic feet or peaks (10). Still, the PPG waveform morphology also carries relevant information that can be exploited, e.g., for arterial stiffness (13, 14) and blood pressure (15) indirect estimation, or early detection of adverse cardiac events (16) or mental disorders signs (17).

Although the PPG signal has proven its potential as a helpful tool in different health domains, its clinical application is still hampered by its poor robustness to several sources of inaccuracy (18), which can be detrimental to the PPG signal quality or misleading for the interpretation of the extracted parameters (19). This limitation is particularly emphasized in the real world, where the monitored subjects conduct their daily-life activities and are no longer in a controlled environment like in laboratory experiments.

The recent article of Fine et al. (18) offers a detailed review of the main factors that influence the PPG signal and its extracted features. If not adequately controlled, these factors may preclude the development of reliable PPG-based applications. Specifically, Fine et al. grouped the sources of inaccuracy in three categories: external perturbations, variations within and across individuals, and physiology. As an external perturbation, physical movement is the primary source of inaccuracy in the PPG signal; on the one hand, it is well-recognized that physical movement leads to signal quality deterioration (19); on the other hand, it also influences the cardiovascular system, and in turn, the PPG morphology, inducing temporary changes, as the cardiovascular system must adapt to the physical stress (20). Another external source of inaccuracy is given by the contact pressure between the PPG sensor and the skin, significantly influencing the quality and the morphology of the recorded signal (21, 22). Individual subject variations can also play a role in modifying PPG signal quality and morphology. For example, it is well-known that the dicrotic notch is less visible as age increases (23), making systolic and diastolic waves less pronounced. This factor can lower the probability of finding Diagnostic quality pulses in older subjects, limiting the possibility of conducting an in-depth morphological analysis. Also, it is well-known that sex can affect the cardiovascular system, thus, in turn, the PPG morphology (24). Finally, the health state can also have an impact on PPG, even in those cases in which the pathology is not closely related to the cardiovascular system. For example, some recent studies demonstrated the link between cancer and cardiovascular alterations, which can origin both from the pathology itself or from cancer treatment (25). Several studies have already investigated the association between cancer and heart rate variability, pointing out significant parameters' alterations in the oncological population (26, 27), also by using PPG (28). In addition, some studies also revealed an impact of cancer (29) and related therapies (30) on PPG signal waveform. From this evidence, it is clear that a PPG-based system that is agnostic to the health state of the subject may lead to misinterpretation of the extracted parameters, failing its primary goal of providing continuous accurate monitoring (18).

Whatever the final application, all these factors, if not adequately controlled, can have a dual negative effect: on the one hand, they can have a different impact on PPG signal quality, hindering the extraction of meaningful PPG features (e.g., a small amount of Diagnostic pulses prevents a reliable, in-depth morphological analysis), and, on the other hand, they can act as confounding factors, invalidating the interpretation and the reliability of the parameters extracted from the PPG morphology. Therefore, to obtain a “true health monitoring” (18) PPG-based application, a proper characterization of these factors is crucial.

This work aims to characterize the impact of these factors, namely physical activity, health state, age, and sex, both on PPG signal quality and PPG waveform parameters. We used a convenience sample of 31 participants, 19 healthy subjects, and 12 oncological patients, monitored in a real-world scenario. For each subject, we selected an equal number of PPG pulses for four different physical activity ranges (estimated by the accelerometer data) and labeled them with a quality level. Firstly, we evaluated the quality levels distribution based on the factors above. We then extracted six morphological parameters and appraised their behavior in relation to physical activity, health state, age, and sex.

## Materials and Methods

### Dataset

**Figure 2:**
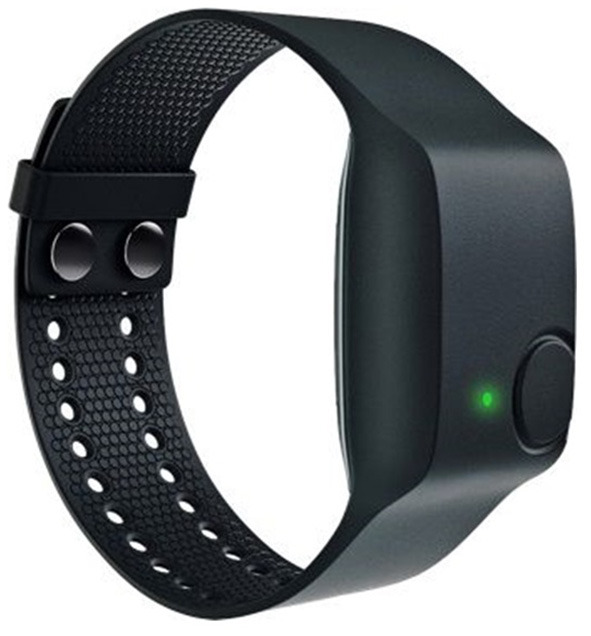
Empatica E4 wristband.

Thirty-one subjects (19 healthy subjects and 12 oncological patients) were monitored for 24 h with the Empatica E4 wristband (Figure 2), a medical-grade wearable device that records several physiological signals, including:

- PPG signal: the sensor is equipped with four light sources (two green, two red) and two matched photodetectors, with a sampling frequency of 64 Hz.
- Accelerometer (ACC) signal: the sensor consists of a tri-axial accelerometer with a ±2 g range and a sampling frequency *fs*_*ACC*_ = 32 Hz.

Subjects were instructed to conduct their daily routine activities and remove the E4 wristband while showering. They were also asked to provide their age and sex.

The study was conducted according to the declaration of Helsinki, and each subject signed informed consent before participating in the study. The two datasets (healthy subjects and oncological patients) belong to two different studies: (1) healthy subjects' recordings were obtained from an internal data collection campaign involving researchers and students at the Personal Health Systems Lab of the University of Bologna; (2) oncological patients' recordings come from an interventional study approved by the Local Ethical Committee (Area Vasta Emilia Centro, Bologna, Italy; approval n° 542-2019-OSS-AUSLBO) (31).

### Signal Processing

PPG signals were filtered by applying a second-order Butterworth band-pass filter, with cut-off frequencies of 0.5 and 12 Hz (32), and consequently divided into PPG pulses by applying the algorithm by Elgendi et al. (33) for systolic feet detection. Each pulse was then normalized with the z-score procedure (34), and the baseline (i.e., systolic feet values) was set to zero.

ACC signals' components were firstly resampled at 64 Hz (*fs*_*ACC*−*RES*_) with linear interpolation to reach the same PPG sampling frequency and then filtered by applying a fourth-order band-pass filter, with cut-off frequencies of 0.025 and 10 Hz (35, 36). We then calculated the ACC vector magnitude for each sample *j* as:


(1) $$\begin{array}{r} A_{j}=\sqrt{ACC_{x_{j}}^{2}+ACC_{y_{j}}^{2}+ACC_{z_{j}}^{2}} \end{array}$$

The Activity Index (*A*_*ind*_) was estimated using the algorithm of Lin et al. (37):

- Standard deviation of *A*_*j*_ for *T* = 5 *s* epochs: (1)

(2) $$\begin{array}{r} \sigma =\sqrt{\frac{1}{N}\overset{N}{\underset{j\;=\;1}{\sum }}{\left(A_{j}\;-\;\mu \right)}^{2}} \end{array}$$

where


$$\begin{array}{r} \mu =\frac{1}{N}\left(A_{1}+A_{2}+\ldots +A_{N}\right)\\ N=T^{*}fs_{ACC-RES} \end{array}$$

Minute-wise *A*_*ind*_:


(3) $$\begin{array}{r} A_{ind}\;=\overset{M}{\underset{k\;=\;1}{\sum }}\sigma_{k} \end{array}$$

where *M* is set to 12 to obtain a minute-wise *A*_*ind*_ by summing 12 epochs.

- *A*_*ind*_ resampling at 64 Hz with linear interpolation.

Once we estimated the *A*_*ind*_ for each recording, we defined four activity ranges (AR) based on the quartile values of all the activity indices.

Each PPG pulse was then associated with an *A*_*ind*_ value as a final signal processing step.

### PPG Pulse Classification

We randomly selected a subset of 100 PPG pulses for each AR within each subject's recording, thus obtaining 400 pulses for each subject (12,400 PPG pulses in total). We chose 400 pulses per subject as a good trade-off between the need to have a representative sample of all pulses and the clinical effort needed to evaluate and label them. It is also in line with previous studies (38, 39). Three independent raters (a cardiologist and two biomedical engineers, all three experts in cardiovascular signals) assigned to each pulse one of the following quality levels:

- Bad (B)
  Systolic and diastolic peaks cannot be distinguished from noise, so the pulse is not suitable for analysis;
- Fair (F)
  The systolic peak is clearly detectable, and the diastolic peak is not so that the pulse can be used for a heart rate estimate and a basic morphological analysis;
- Excellent (E)
  Both the systolic and diastolic peaks can be clearly detected so that the pulse can be used for heart rate estimate and in-depth morphological analysis.

**Figure 3:**
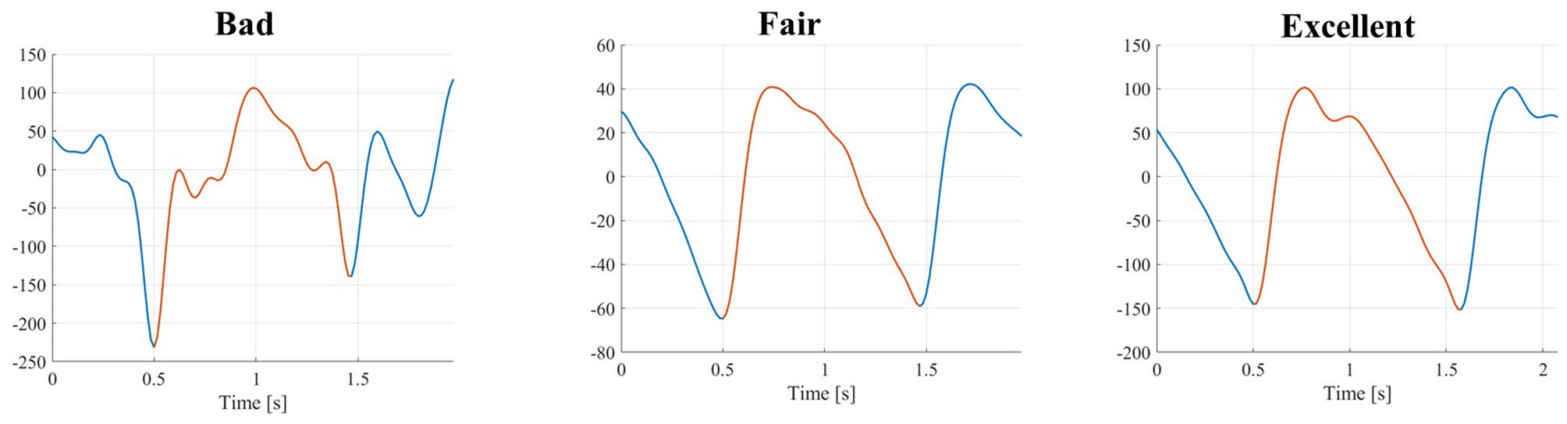
PPG pulses with three different quality levels (from left to right): bad, fair, and excellent.

An example of the three different quality levels is presented in Figure 3.

We adopted a majority voting approach to determine the level if only two raters agreed. If there was no agreement among raters, the pulse was automatically labeled as B.

Based on these quality levels, Basic PPG pulses were obtained as the union between F and E pulses, while the Diagnostic PPG pulses coincide with the only E pulses (12).

### PPG Waveform Parameters Estimation

We estimated six PPG parameters only on those PPG pulses suitable for analysis (i.e., Basic and Diagnostic pulses), thus discarding the B quality pulses. For both Basic and Diagnostic pulses, the systolic peak is the highest value found using the Matlab *findpeak* function within each PPG pulse. For Diagnostic pulses, the same Matlab *findpeak* function is applied, and the diastolic peak is found as the second-highest value.

From Basic pulses, we estimated:

- Rise Time (*RT*) [s]: time between the systolic foot and the subsequent systolic peak (40);
- Pulse Time (*PT*) [s]: time between two consecutive systolic feet (10, 39);
- Pulse Amplitude (*PA*) [a.u.]: height of the systolic peak, with the previous systolic foot as the reference (38).

From Diagnostic pulses, we estimated:

- Reflection index (*RI*) [%]: ratio between diastolic and systolic amplitude (10, 39);
- Delta T (Δ*T*) [s]: time lag between systolic peak and the subsequent diastolic peak (41);
- Diastolic Amplitude (*DA*) [a.u.]: height of the diastolic peak, with the previous systolic foot as the reference (10).

**Figure 4:**
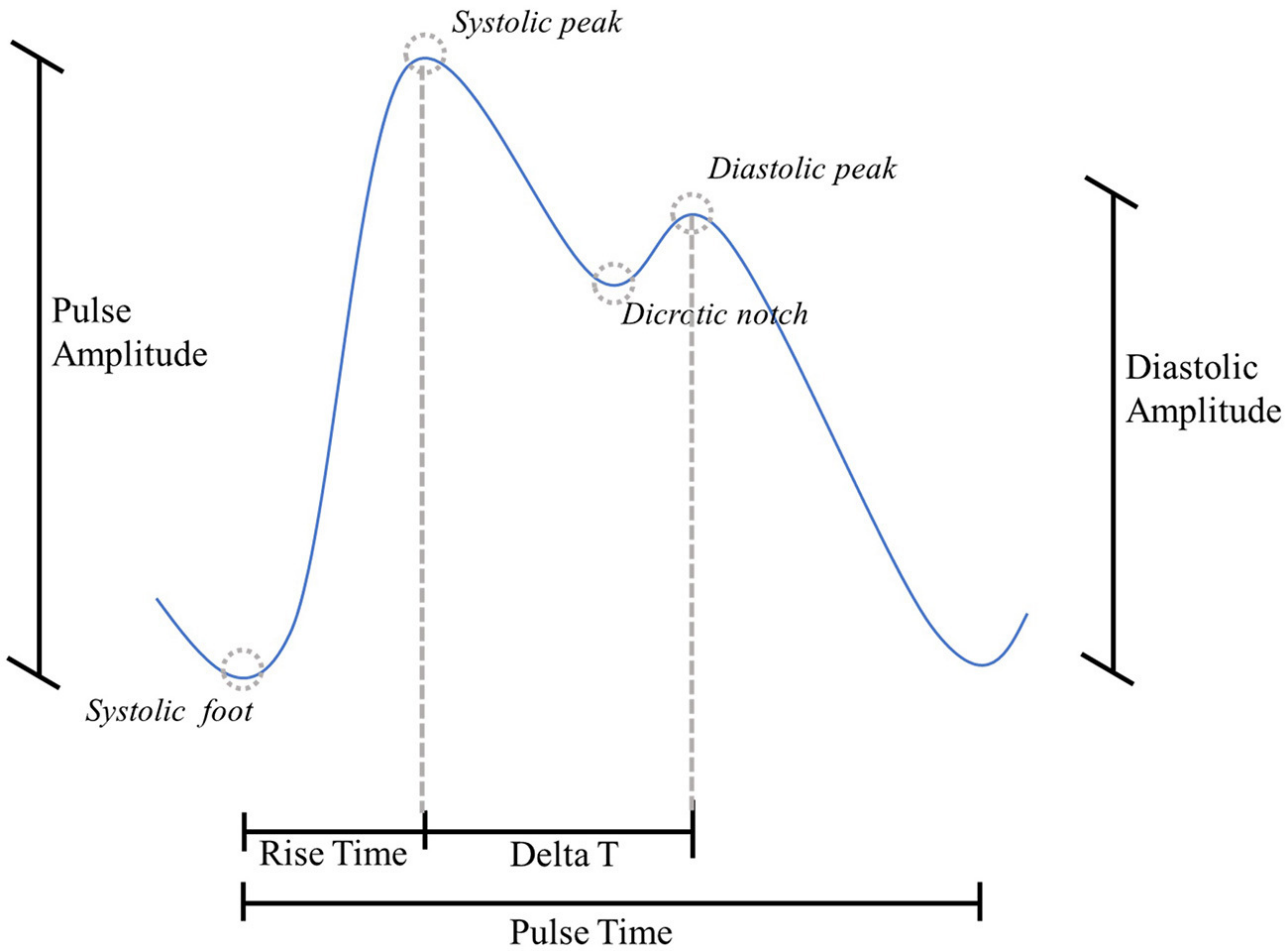
PPG morphology parameters.

A graphical representation of the PPG above parameters is proposed in Figure 4.

### Statistical Analysis

To qualitatively assess the impact of physical activity and health state on PPG signal quality, we evaluated the distribution of quality levels among the four AR and throughout the 24 h separately for healthy and oncological subjects. To statistically assess the impact of physical activity, health state, age, and sex on PPG signal quality, we implemented a multinomial logistic regression (MLR) model. MLR is used to predict the relative probability of being in one category compared to being in a reference category, obtained with a linear combination of predictor variables that can be continuous or categorical. The logit function is usually employed as a link function for MLR models. Setting the *K-*th category as a reference, the MLR can be written as (42):

(4) $$\begin{array}{r} \pi_{j}=\mathrm{Pr}\left(y=j|\text{x}\right)=\;\frac{\exp\left({\beta_{\text{j}}}^{\text{T}}\text{x}\right)}{1+\sum_{k=1}^{K-1}\exp\left({\beta_{\text{k}}}^{\text{T}}\text{x}\right)} \end{array}$$

where π_*j*_ is the *j-th* category membership probability against the reference category *K*, ***β***_**j**_ is the regression coefficients vector, and **x** is the regressors vector. We set *A*_*ind*_ (continuous variable), health state (dichotomous variable, 0 = healthy subject, 1 = oncological subject), age (continuous variable), and sex (dichotomous variable, 0 = male, 1 = female) as regressors vector.

To evaluate the influence of physical activity, health state, age, and sex on PPG waveform parameters, we fitted each PPG parameter with a Generalized Linear Mixed-Effects Model (GLMM). GLMMs extend the generalized linear models, allowing to model both fixed and random effects. A simple Linear Mixed-Effects model can be written as (43):

(5) $$\begin{array}{r} E\left(y|\text{X},\text{Z}\right)=\text{X}\beta +\text{Z}\text{u} \end{array}$$

where **X** is the matrix of the fixed effects, ***β*** is the vector of fixed effects regression coefficients, **Z** is the matrix of the random effects, **u** is the vector of random effects coefficients, and *E*(*y*|**X**, **Z**) is the expected outcome variable conditional on **X** and **Z**. In a “Generalized” Linear Mixed-Effects Model, the outcome variable can have a non-normal distribution so that a GLMM can be expressed as:

(6) $$\begin{array}{r} g\left(E\left(y|\text{X},\text{Z}\right)\right)=\text{X}\beta +\text{Z}\text{u} \end{array}$$

where *g*(•) is the link function for the outcome variable. The link function maps the relationship between the conditional expected outcome and the linear combination of the predictors. There is an associated canonical link function for each distribution of the outcome variable.

GLMMs are particularly useful when data samples are non-independent, such as, e.g., in a hierarchical structure (i.e., different instances coming from a single subject) (44). We fitted one GLMMs for each of the six PPG parameters, using the Basic pulses to determine *RT, PT*, and *PA*, and the Diagnostic pulses to determine *RI*, Δ*T*, and *DA*. We set the PPG parameter as the outcome variable, the four factors as the fixed effects, while the “subject” variable was set as the random effect to consider the inter-subject variability. We tested three different distributions for the GLMMs (and the respective link functions): normal (identity), gamma (negative inverse), and inverse Gaussian (inverse squared), the last two suitable to model non-negative outcome variables. Table 1 presents the three distributions and the respective link functions. We then chose the best model based on the Akaike Information Criterion (AIC) (45) and evaluated the results, both for fixed and random effects (46). We performed a marginal F-test to determine the significance of single fixed-effects coefficients. To test the significance of the random effects, we evaluated the 95% standard deviation's confidence interval as the estimated covariance parameter for the random effects (i.e., “subject”): if the interval does not contain 0, the random effects are significant at the 5% significance level. The analyses were carried out on Matlab 2021b (47).

**Table 1:** Distribution of the outcome variable and respective link function. ***μ*** is expected value of y (outcome) conditional on x (predictors).

**Distribution**	**Link function**
Normal	*g*(μ) = μ
Gamma	*g*(μ) = −μ^−1^
Inverse Gaussian	*g*(μ) = μ^−2^

## Results

### Descriptive Statistics

We analyzed PPG recordings from 31 subjects, 19 healthy subjects and 12 oncological patients (one bone and soft tissue, four gastrointestinal, two genital tract, one endocrine, one hematological, two breast, one urinary). The demographics of the sample are reported in Table 2. The average recording length was 26:50 (±05:51) h.

**Table 2:** Demographics of the sample.

	**All**	**Healthy**	**Oncological**
Sample Size	31	19	12
Age [years]	37 ± 13.8	29.2 ± 7.1	49.5 ± 12.8
Sex	15 M, 16 F	13 M, 6 F	2 M, 10 F

Quartile values of the *A*_*ind*_ distribution were *Q*_1_ = 0.04, *Q*_2_ = 0.41, *Q*_3_ = 1.32, with a maximum value of 6.75. According to the classification made by Lin et al. (37), the four ARs correspond, respectively to rest/sleep, rest/sleep/sedentary, light activity, and moderate activity.

For the 12,400 randomly selected pulses, the three independent raters agreed on 86% of the labels. By applying a majority voting approach, we obtained the following labels distributions: 5,962 (48.1%) B pulses, 4,612 (37.2%) F pulses, and 1,826 (14.7%) E pulses. Table 3 reports the distribution of quality levels for each subject.

**Table 3:** Distribution of quality levels among healthy subjects and oncological patients.

	**Subject**	**B**	**F**	**E**
	1	251	81	68
	2	157	203	40
	3	133	255	12
	4	227	45	128
	5	214	57	129
	6	239	85	76
	7	136	164	100
	8	144	192	64
Healthy subjects	9	207	155	38
	10	208	84	108
	11	276	60	64
	12	170	128	102
	13	139	197	64
	14	231	113	56
	15	103	185	112
	16	124	239	37
	17	217	106	77
	18	316	31	53
	19	126	59	215
	1	203	197	0
	2	147	242	11
	3	160	239	1
	4	229	168	3
	5	222	171	7
Oncological patients	6	206	147	47
	7	205	123	72
	8	194	183	23
	9	127	248	25
	10	217	173	10
	11	189	162	49
	12	245	120	35

*B, Bad; F, Fair; E, Excellent*.

### Impact on PPG Pulses Quality

**Figure 5:**
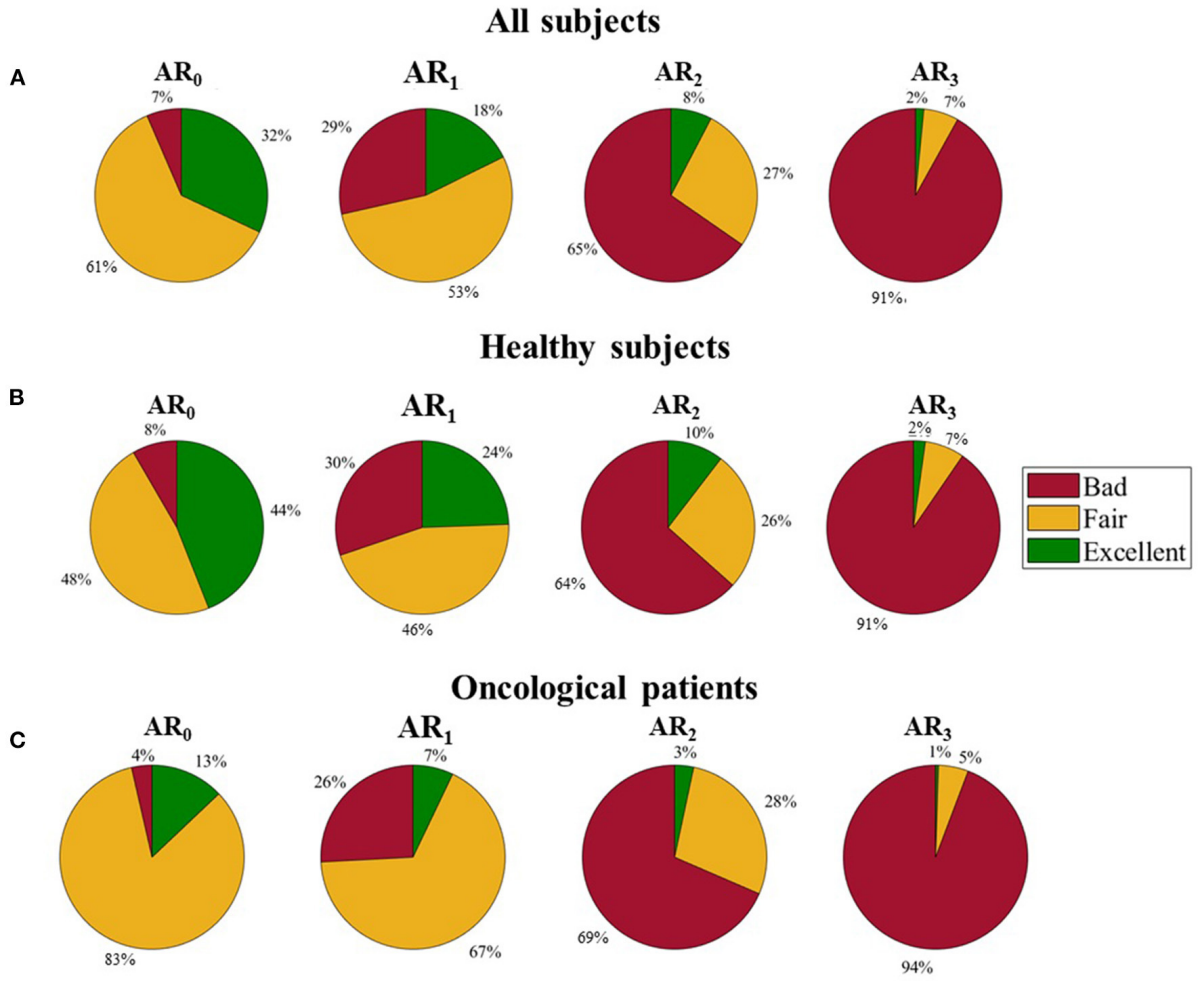
Distribution of the three quality levels among different activity ranges. **(A)** All subjects, **(B)** Healthy subjects, and **(C)** Oncological patients.

We evaluated the distribution of the three quality levels among the four ARs. As can be seen in Figure 5, panel a, the percentage of B pulses rises as the physical activity increases (ranging from 7% in AR_0_ to 92% in AR_3_), while the percentage of F and E pulses decreases (from 62% in AR_0_ to 7% in AR_3_ for F pulses; from 32% in AR_0_ to 2% in AR_3_ for E pulses).

By analyzing separately healthy and oncological subjects, the different distribution of the three quality levels appears evident (see Figure 5, panels b and c): oncological patients present a lower percentage of E pulses in all the ARs and a higher percentage of F pulses in the lowest ARs (84 and 67% for oncological patients against 48 and 45% for healthy subjects in AR_0_ and AR_1_, respectively).

**Figure 6:**
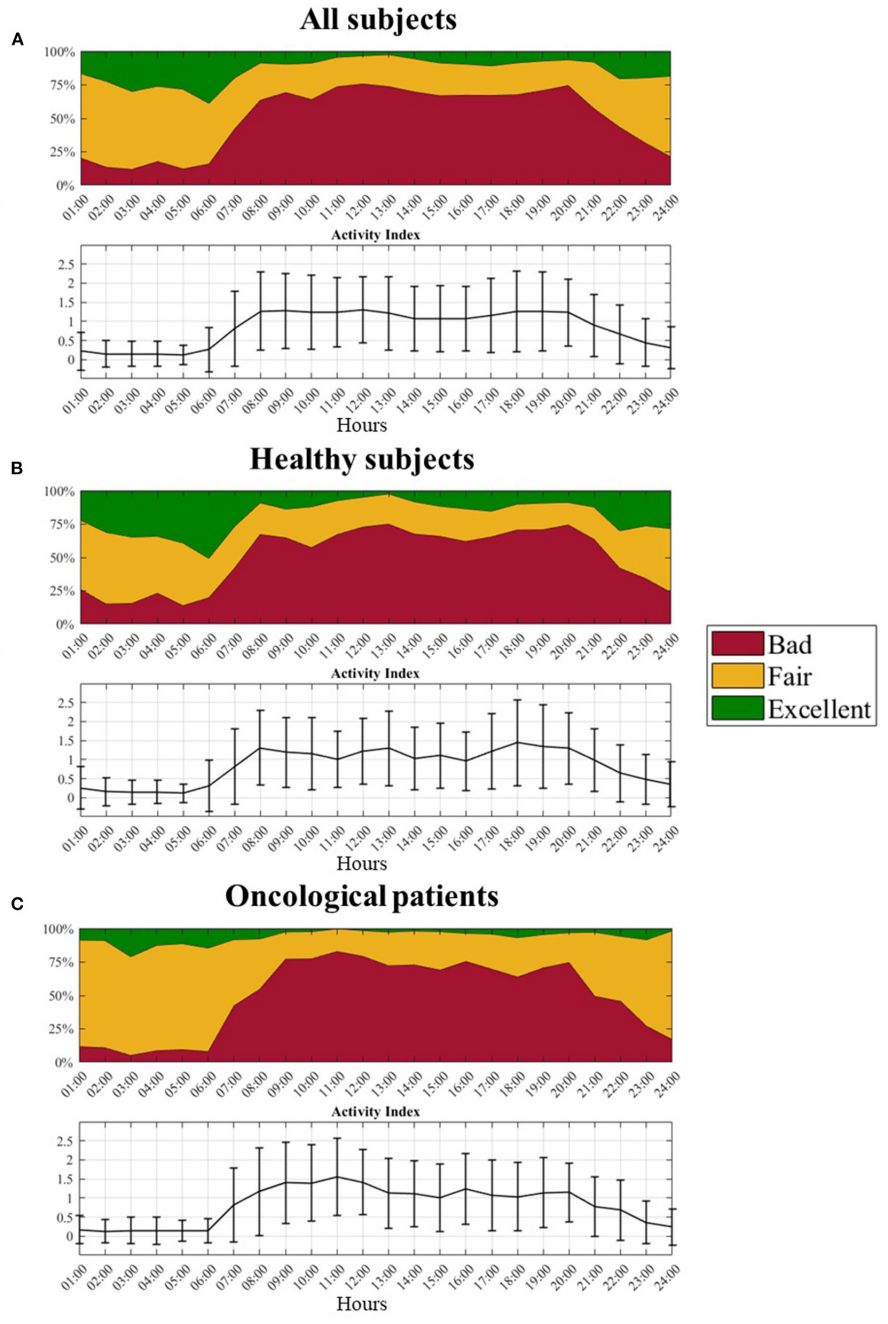
Distribution of the three quality levels and related activity index profile over the 24 hours. **(A)** All subjects, **(B)** Healthy subjects, and **(C)** Oncological patients.

A graphical representation of the quality levels throughout the 24 h is provided in Figure 6, together with the *A*_*ind*_ values. The figure shows the percentage of the different quality levels during each hour. A higher percentage of F and E pulses can be found during the night when the *A*_*ind*_ values are lower both evaluating the whole dataset (panel a) and dividing it into healthy (panel b) and oncological subjects (panel c). During the night, oncological patients present a lower number of B pulses (around 10%) compared to healthy subjects (around 20%).

From the MLR model, we obtained the results reported in Table 4, setting the B quality level as the reference category. We present here the ***β*** coefficients for each regressor (i.e., *A*_*ind*_, health state, age, and sex), whose interpretation is the following: positive ***β*** coefficients represent a direct association between the regressor and the probability of belonging to that category compared to the reference one; higher values mean a stronger relationship between the regressor and the probability of belonging to that category compared to the reference one. *A*_*ind*_ has a significant impact on the relative probabilities (with respect to the B quality level) for both F and E quality levels: as *A*_*ind*_ increases, the relative probability of belonging to F and E quality levels decreases. Health state significantly influences the relative probability of having F and E quality pulses: oncological patients have an increased relative probability of having F pulses, while there is a lower relative probability for the same population of having E pulses. Finally, age significantly influences the relative probabilities of F and E quality levels: the coefficient has a positive value (0.03) for F quality level and a negative value (−0.0088) for E quality level. This means a higher relative probability of having F pulses and a lower relative probability of having E pulses as the age increases. Regarding sex, female subjects have an increased relative probability of having F (0.84) and E (0.48) pulses compared to males.

**Table 4:** Multinomial logistic regression coefficients.

	Fair^*^		Excellent^*^	
	**Estimate**	***p*-value**	**Estimate**	***p*-value**
***A*_*ind*_**	−2.31	0	−3.03	0
**HealthState (Oncological)**	0.15	0.04	−0.87	0
**Age**	0.03	0	−0.009	0.02
**Sex (Female)**	0.84	0	0.48	0
**Intercept**	−0.42	0.0001	0.79	0

* *Against Bad quality level (set as reference category)*.

### PPG Waveform Parameters

After grouping pulses into Basic (F+E) and Diagnostic (E) pulses, we obtained the following proportions:

- 6,438 Basic pulses, 3,944 from healthy subjects (61.3%), and 2,494 from oncological subjects (38.7%)
- 1,826 Diagnostic pulses, 1,540 from healthy subjects (84.3%), and 286 from oncological subjects (15.7%)

We fitted six different GLMMs, one for each PPG parameter, using the Basic pulses to determine *RT, PT*, and *PA*, and the Diagnostic pulses to determine *RI*, Δ*T*, and *DA*. Table 5 shows the AIC values for all the six models using normal, gamma, and inverse Gaussian distributions. Four out of six models were best fitted with a normal distribution (*RT, PT, RI*, and Δ*T*), while two (*PA* and *DA*) were best fitted with an inverse gaussian distribution. The interpretation of ***β*** coefficients for the two distributions (and the related link functions) is the following: for the normal distribution (and identity link function), positive coefficients indicate that the outcome variable increases if the predictor increases; for the inverse Gaussian distribution (and inverse squared link function), positive coefficients indicate that the outcome variable increases if the predictor decreases.

**Table 5:** Akaike Information Criterion (AIC) for different models.

**AIC**	**Normal**	**Gamma**	**Inverse Gaussian**
RT	–**18,186**	15,611	41,620
PT	–**10,034**	−79,912	2,326
PA	−1,337	−30,577	–**36,263**
RI	–**2863.2**	−729	2,927
ΔT	–**6,183**	4,067	11,774
DA	1,491	−4,819	–**5,389**

*In bold the lowest AIC number for each outcome variable, corresponding to the best fitting model*.

**Figure 7:**
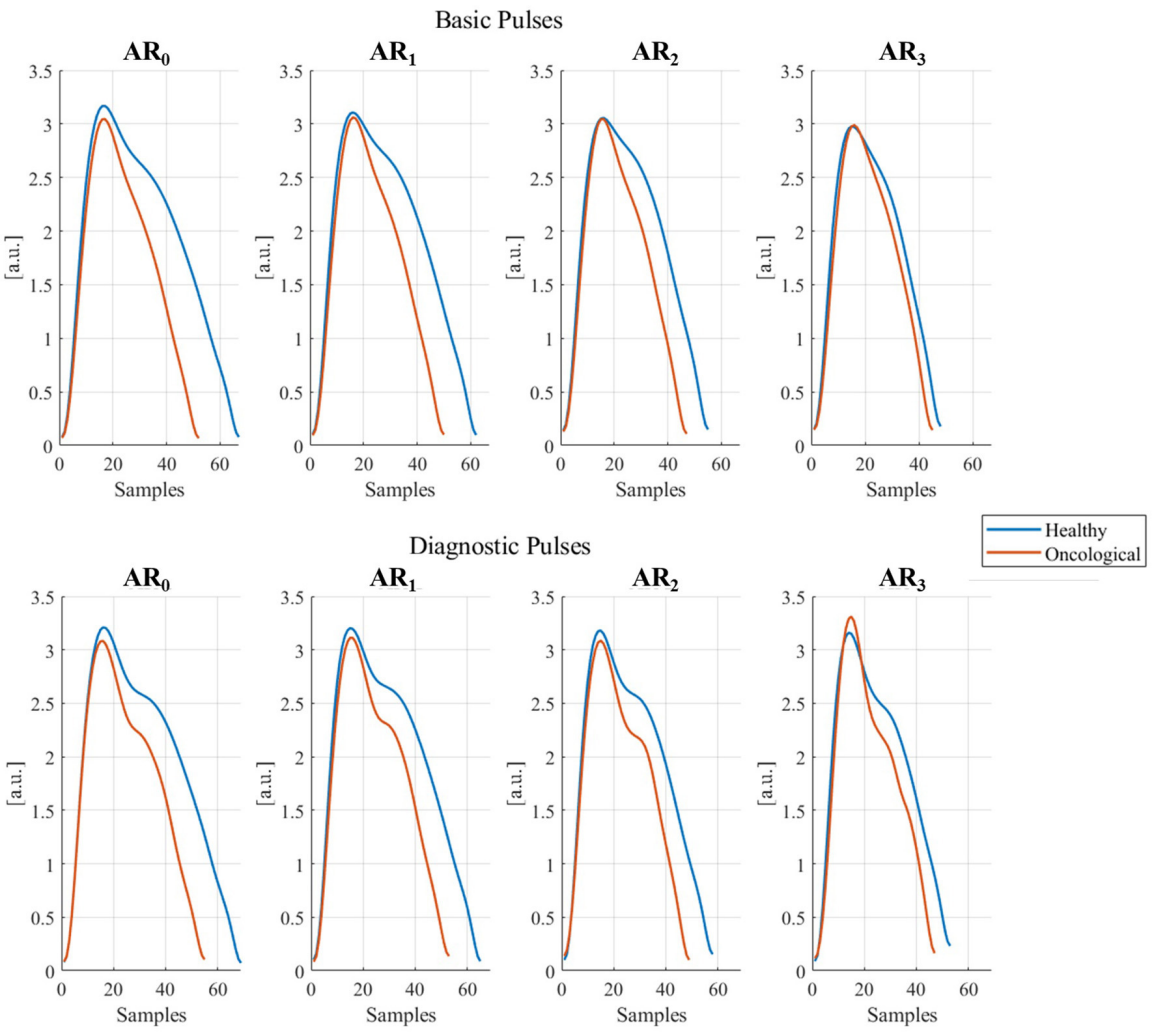
Basic and diagnostic pulses in different activity ranges (*AR*_i_, i = 1, …, 3) in healthy and oncological subjects. The represented pulses were obtained as the mean of all collected pulses for each AR and dividing them for healthy and oncological subjects.

In Table 6 results from the GLMMs are shown. All PPG parameters, except *RT*, are significantly influenced by physical activity (*A*_*ind*_). Specifically, all the parameters coefficients tend to have lower values as the *A*_*ind*_ increases. The health state significantly influences *PT, PA, RI*, and *DA*: these parameters assume lower coefficient values for oncological patients than healthy subjects. Age significantly influences Δ*T* and *DA*: Δ*T* is shorter as age increases, while *DA* increases with age progression. Sex does not have any significant effect on the analyzed parameters. Since the 95% random-effects confidence intervals for all the PPG parameters do not contain the 0 value, inter-subject variability is significant for all the tested PPG parameters. Figure 7 shows the graphical representation of both Basic and Diagnostic PPG pulses, as the mean of the analyzed pulses, for different ARs and health states.

**Table 6:** Generalized linear mixed effects models.

	**Fixed effects**					**Random effects** **σ(subject)**		
		**Estimate**	**Lower**	**Upper**	**F-Test**	**Estimate**	**Lower**	**Upper**
	***A*_*ind*_**	−0.00	−0.0055	0.001	0.21	0.03	0.03	0.04
	**Health state (Oncological)**	−0.01	−0.03	0.005	0.14			
***RT* ^§^**	**Age**	0.0005	−0.0008	0.002	0.47			
	**Sex (Female)**	−0.004	−0.02	0.01	0.61			
	**Intercept [s]**	0.24	0.19	0.30	0			
	***A*_*ind*_**	−0.12	−0.13	−0.12	**0**	0.12	0.09	0.15
	**HealthState (Oncological)**	−0.12	−0.18	−0.05	**0.0002**			
***PT* ^§^**	**Age**	−0.0005	−0.005	0.004	0.85			
	**Sex (Female)**	−0.03	−0.08	0.02	0.25			
	**Intercept [s]**	0.94	0.76	1.11	0			
	***A*_*ind*_**	0.005	0.004	0.006	**0**	0.005	0.004	0.006
	**HealthState (Oncological)**	0.003	0.0005	0.006	**0.02**			
***PA*** ^*^	**Age**	0.0001	−0.0001	0.0003	0.39			
	**Sex (Female)**	0.0004	−0.002	0.002	0.71			
	**Intercept [a.u.]**	0.10	0.09	0.11	0			
	***A*_*ind*_**	−0.02	−0.04	−0.01	**0.0003**	0.09	0.07	0.01
	**HealthState- (Oncological)**	−0.06	−0.11	−0.02	**0.008**			
***RI* ^§^**	**Age**	0.003	−0.0007	0.006	0.12			
	**Sex (Female)**	0.02	−0.01	0.06	0.25			
	**Intercept []**	0.67	0.53	0.80	0			
	***A*_*ind*_**	−0.01	−0.02	−0.01	**0**	0.03	0.03	0.04
	**HealthState (Oncological)**	0.005	−0.01	0.02	0.58			
**Δ*T*^§^**	**Age**	−0.001	−0.003	−0.0002	**0.03**			
	**Sex (Female)**	−0.001	−0.02	0.01	0.84			
	**Intercept [s]**	0.30	0.24	0.35	0			
	***A*_*ind*_**	0.01	0.01	0.02	**0.0001**	0.04	0.03	0.06
	**HealthState (Oncological)**	0.04	0.02	0.05	**0.0004**			
***DA*** ^*^	**Age**	−0.002	−0.004	−0.0004	**0.016**			
	**Sex (Female)**	−0.01	−0.03	0.004	0.11			
	**Intercept [a.u.]**	0.26	0.19	0.33	0			

§ *Normal distribution and identity link function*.

* *Inverse Gaussian distribution and inverse squared link function*.

*RT, Rise Time; PT, Pulse Time; PA, Pulse Amplitude; RI, Reflection Index; ΔT, delta T; DA, Diastolic Amplitude; A_ind_, Activity Index*.

*In bold the p-values lower than 0.05*.

## Discussion

This study assessed the impact of several sources of inaccuracy on PPG signal quality and PPG waveform parameters by using 31 24 h real-world recordings, 19 from healthy subjects and 12 from oncological patients. We randomly selected 400 pulses for each recording, 100 for each physical activity quartile and labeled them into three quality levels. We compared the quality levels distribution among different physical activity ranges throughout the 24 h. We then used a Multinomial Logistic Regression model to quantitatively evaluate the impact of physical activity, health state, age, and sex on PPG signal quality. We finally estimated six PPG parameters only on higher-quality pulses (i.e., Basic and Diagnostic quality) and fitted each of them into a Generalized Linear Mixed Effects model to evaluate their sensitivity to physical activity, health state, age, and sex.

Physical activity is well-recognized as the main cause hindering the clinical application of PPG signals in daily life (48). This study could demonstrate its detrimental effect by comparing the quality levels distribution among different physical activity ranges. As expected, as the physical activity got more intense, the percentage of higher quality pulses (i.e., F and E) got lower. Similar results were also obtained from the MLR model's fitting, confirming a lower relative probability of having F and E pulses against B pulses as the physical activity increased. Reliable information can thus be gathered in case of low physical activity, for example, when the subject is at rest or in sedentary conditions, corresponding to AR_0_ and AR_1_, in agreement with previous literature (49). As also Pradhan et al. (50) highlighted, the best data quality could be obtained during the night when the subjects were likely to be asleep. However, a prodromic signal quality analysis appears necessary to obtain reliable data from PPG signal processing.

Another interesting aspect is the different quality distribution obtained by analyzing pulses of healthy and oncological subjects separately. The latter group showed a lower percentage of E pulses than the former in the lowest ARs, and concurrently a higher percentage of F pulses. In addition, cancer subjects were shown to have fewer negative pulses than healthy subjects. This could mean that the pathological condition (cancer in this case) does not affect the quality of PPG signal to such an extent that it completely loses its information content (i.e., B pulses) but rather negatively affects the morphology of PPG pulses by losing definition (i.e., F pulses). The MLR model confirms that for oncological patients and as the age increases, i) the relative probability of having F pulses (compared to B pulses) increases, and ii) the relative probability of having E pluses (compared to B pulses) decreases (positive coefficients). The lower probability of finding E pulses can be ascribed either to a deterioration of the cardiovascular health state because of the pathology itself and the related therapy (51) or to the higher age of the oncological patients compared to the healthy subjects (41), as can be seen in Table 1.

Sex had a significant impact on PPG pulses quality. The relative probability of having F and E pulses against B pulses was considerably higher for female subjects than males. Previous studies reported significant sex-based differences in the pulse transit time, that is, the time between the R peak recorded through the electrocardiogram and the consecutive PPG cycle (24), and it is well-known that the cardiovascular system differs between women and men, both in physiological and in pathological conditions (52). A previous study has already found that commercial smartwatches are less accurate, for heart rate measurement only, for men than for women (53). This difference could be due to the different skin thickness, higher for males than females (54): PPG sensor light has to pass through a larger thickness in male subjects, which could then lead to a deterioration in the PPG signal quality.

From the analysis of PPG waveform parameters, we found that all parameters, except *RT*, were significantly influenced by physical activity, lowering their values. This result is Two-fold: rise time RT can be used as a parameter independent of physical activity; conversely, other parameters must be interpreted in light of current physical activity levels. Furthermore, pathological states should also be considered when interpreting *PT, PA, RI*, and *DA*, negatively affecting their values. Finally, age had a significant impact only on Δ*T* and *DA*: as the age increases, the former assumes lower values while the latter increases. These findings agree with previous literature. Specifically, other authors have found that aging causes a reduction in the time between systolic and diastolic peaks (Δ*T*) (10, 55) and an increase in the diastolic amplitude (*DA*) (10, 39), mainly due to an increased arterial stiffness (56). Since the diastolic peak depends on the reflection of the pressure wave on artery walls, a loss of elasticity (i.e., increased arterial stiffness) brings to a quicker and more intense wave reflection (57).

The PPG signal quality analysis results recommendusing features extracted from a basic morphological analysis (i.e., using Basic quality pulses) rather than from an in-depth morphological analysis (i.e., using Diagnostic quality pulses) in the real-world. This is remarkably advisable if the PPG-based application should be used by subjects at risk of cardiovascular system impairment or deterioration. Unfortunately, several experimental PPG-based applications use features that can be extracted only on Diagnostic quality pulses (58–61), thereby risking malfunctioning in this population, especially in real-world conditions, where the availability of Diagnostic quality pulses is further lowered because of the presence of motion artifacts.

In addition, our results related to the PPG waveform parameters confirmed, as already pointed out by Fine et al. (18), that future PPG-based applications should accurately consider several personal and health-related factors, as these can act as sources of inaccuracy and limit the interpretability and generalizability of the results. PPG sensors undoubtedly have excellent qualities, as they can be easily embedded in wearable devices, are inexpensive, and can collect a variety of vital information. A proper characterization of the various sources of inaccuracy influencing the PPG signal may expand its use in the clinical field, obtaining a powerful tool allowing pervasive and continuous recordings.

Besides the several sources of inaccuracy the PPG can be subjected to, it is worth remembering that different physiological activities can also influence PPG waveform parameters: in particular, for the time parameters (*RT, PT*, Δ*T*), changes in cardiac activity have a significant impact on the timing of the events reflected in the PPG waveform (11); respiration can induce variations affecting both the pulsatile and non-pulsatile components of the PPG signal (62); lastly, PPG is strongly affected by the autonomic nervous system, leading to significant changes especially in the time domain (63).

This study presents some limitations, primarily related to the sample size. As previously pointed out, age significantly differed between healthy and oncological subjects (mean age 29.2 vs. 49.5 years), thus partly overlapping the effects due to age and health state. The range of physical activities gives another limitation: our dataset lacks vigorous activities, based on the classification of Lin et al. (37), although, based on our results, we can speculate that a tiny proportion of pulses in that category could be used for further processing (i.e., labeled as F or E).

We used a convenience sample, investigating the impact of cancer as a pathological state. We intended to raise the attention, by providing quantitative results, on how a pathology that apparently should have no impact on the PPG signal can lead to misinterpretations if not adequately considered. This study can help expand the knowledge about the impact of cancer on PPG, with the double objective of i) controlling the “health state” variable for a general purpose application, and ii) using PPG with a diagnostic and/or prognostic value for the oncological population. The transferability of these results to other pathologies should be investigated further. However, this work can pave the way to future studies aiming at evaluating the influence of different pathologies on PPG.

From a technical point of view, the low PPG sampling frequency (64 Hz) may limit the accuracy of those time-domain parameters with an order of magnitude comparable to the sampling period (0.0156 s), such as *RT* and Δ*T* (mean values equal to 0.26 s and 0.25 s, respectively). In the present study, we obtained 34 different values for *RT* and 27 for Δ*T*, so the analysis can still be considered valid. However, we suggest deepening this aspect by using sensors with a higher sampling frequency.

Future studies can be conducted on larger datasets, with a more heterogeneous sampling by age and physical activity and deepening the effects of other personal and health factors, such as weight and height, or other pathological states.

## Conclusions

This study aimed to evaluate the impact of different sources of inaccuracy both on PPG signal quality and on parameters extracted from the PPG morphology. We used a convenience sample of healthy subjects and oncological patients to assess the impact of physical activity, age, sex, and health state. As expected, we found that a higher percentage of good quality PPG pulses can be found during the night and when the subject is in sedentary conditions. Age, pathological state, and male sex are three factors that lower the probability of finding Diagnostic quality pulses. Regarding the impact of these factors on PPG morphology parameters, physical activity and health state must be considered when interpreting parameter values, while age acts more on those PPG parameters closely related to arterial stiffness. Therefore, it is advisable to conduct further studies on this topic on larger datasets, investigating the effects of different pathological conditions on the PPG signal. Such an approach can help expand the use of PPG-based application, offering a greater robustness and, thus, a more reliable tool for continuous and pervasive monitoring.

## Data Availability Statement

The raw data supporting the conclusions of this article will be made available by the authors, upon reasonable request.

## Ethics Statement

The study was conducted in accordance with the Declaration of Helsinki. A portion of the data come from a study approved by Ethical Committee of Area Vasta Emilia Centro (Bologna, Italy; approval n◦ 542-2019-OSS-AUSLBO). For the rest of the data (healthy subjects), no approval from the local ethical committee was needed.

## Author Contributions

SM conceived and designed the analysis, collected the data, performed the analysis, and interpreted the results. LP and PP verified the analytical methods and contributed to interpret the results. LC contributed to interpret the results and supervised the project. All authors discussed the results and contributed to the final manuscript.

## Conflict of Interest

The authors declare that the research was conducted in the absence of any commercial or financial relationships that could be construed as a potential conflict of interest.

## Publisher's Note

All claims expressed in this article are solely those of the authors and do not necessarily represent those of their affiliated organizations, or those of the publisher, the editors and the reviewers. Any product that may be evaluated in this article, or claim that may be made by its manufacturer, is not guaranteed or endorsed by the publisher.
